# Do the venous blood samples replicate malaria parasite densities found in capillary blood? A field study performed in naturally-infected asymptomatic children in Cameroon

**DOI:** 10.1186/s12936-017-1978-6

**Published:** 2017-08-17

**Authors:** Maurice M. Sandeu, Albert N. Bayibéki, Majoline T. Tchioffo, Luc Abate, Geoffrey Gimonneau, Parfait H. Awono-Ambéné, Sandrine E. Nsango, Diadier Diallo, Antoine Berry, Gaétan Texier, Isabelle Morlais

**Affiliations:** 1 Laboratoire de Recherche sur le Paludisme, Organisation de Coordination pour la lutte contre les Endémies en Afrique Centrale, BP288, Yaoundé, Cameroon; 2 UMR MIVEGEC, Institut de Recherche pour le Développement, 911 Avenue Agropolis, BP64501, 34394 Montpellier Cedex, France; 3 Université de Douala, Faculté de Médecine et des Sciences Pharmaceutiques, BP2701 Douala, Cameroon; 4 PATH Malaria Vaccine Initiative, Washington, DC 20001 USA; 5 Centre de Physiopathologie de Toulouse Purpan, INSERM U1043, CNRS, UMR5282, Université de Toulouse III, BP 3028, 31024 Toulouse Cedex 03, France; 6 Centre Hospitalier Universitaire de Toulouse, TSA 40031, 31059 Toulouse, France; 7 Centre d’épidémiologie et de santé publique des armées, 111 avenue de la Corse, BP40026, 13568 Marseille Cedex 02, France; 8 UMR 912-SESSTIM-INSERM/IRD, Université Aix-Marseille, 27 bd Jean Moulin, 13385 Marseille Cedex 05, France

**Keywords:** *Plasmodium falciparum*, Membrane feeding, Blood source, Malaria, Cameroon

## Abstract

**Background:**

The measure of new drug- or vaccine-based approaches for malaria control is based on direct membrane feeding assays (DMFAs) where gametocyte-infected blood samples are offered to mosquitoes through an artificial feeder system. Gametocyte donors are identified by the microscopic detection and quantification of malaria blood stages on blood films prepared using either capillary or venous blood. However, parasites are known to sequester in the microvasculature and this phenomenon may alter accurate detection of parasites in blood films. The blood source may then impact the success of mosquito feeding experiments and investigations are needed for the implementation of DMFAs under natural conditions.

**Methods:**

Thick blood smears were prepared from blood obtained from asymptomatic children attending primary schools in the vicinity of Mfou (Cameroon) over four transmission seasons. Parasite densities were determined microscopically from capillary and venous blood for 137 naturally-infected gametocyte carriers. The effect of the blood source on gametocyte and asexual stage densities was then assessed by fitting cumulative link mixed models (CLMM). DMFAs were performed to compare the infectiousness of gametocytes from the different blood sources to mosquitoes.

**Results:**

Prevalence of *Plasmodium falciparum* asexual stages among asymptomatic children aged from 4 to 15 years was 51.8% (2116/4087). The overall prevalence of *P. falciparum* gametocyte carriage was 8.9% and varied from one school to another. No difference in the density of gametocyte and asexual stages was found between capillary and venous blood. Attempts to perform DMFAs with capillary blood failed.

**Conclusions:**

*Plasmodium falciparum* malaria parasite densities do not differ between capillary and venous blood in asymptomatic subjects for both gametocyte and trophozoite stages. This finding suggests that the blood source should not interfere with transmission efficiency in DMFAs.

**Electronic supplementary material:**

The online version of this article (doi:10.1186/s12936-017-1978-6) contains supplementary material, which is available to authorized users.

## Background

Despite recent progress towards disease control, malaria continues to affect 212 million people and kill 429,000 persons per year [1]. In sub-Saharan Africa, *Plasmodium falciparum* remains the most important threat, accounting for most of the malaria mortality [2, 3]. Current control strategies aim at reducing malaria transmission through vector control interventions and appropriate diagnosis and treatment. There is currently no effective vaccine for malaria. The most advanced candidate, the RTS,S, has shown relative low efficacy and WHO has recommended pilot implementations before its deployment [4–6]. Several other vaccine candidates are under development and among them transmission-blocking vaccines (TBVs) that target *Plasmodium* parasites within the mosquito, are in pre-clinical trials [7, 8]. The efficacy of TBVs at reducing or blocking transmission is measured by the reduction of the oocyst intensity and infection prevalence in mosquitoes through membrane feeding assays (MFAs) [8, 9].

In malaria endemic areas, transmission from human to mosquito occurs when a female *Anopheles* mosquito ingests gametocytes, the sexual stages, from peripheral blood while feeding. Thus, gametocytes represent a crucial stage for transmission-reducing interventions (TRIs). However, transmissibility of gametocytes from naturally infected donors is complex and relies on multiple factors from the host, the parasite and the mosquito [10–12]. In a meta-analysis of previous studies, DMFAs were shown suitable for the evaluation of gametocyte infectiousness and different aspects that require optimization for larger scale screening of TBVs were discussed [11]. Differences in the gametocyte concentration or maturity between different blood compartments were pointed out as a putative source of variation in DMFAs.

In the present study, *P. falciparum* parasite densities, trophozoites (ring stages) and gametocytes, were compared between capillary and venous blood samples from naturally-infected gametocyte carriers identified among pupils attending primary schools in Cameroon.

## Methods

### Ethics statement

All procedures involving human subjects used in this study were approved by the Cameroonian national ethics committee (statements 2013/02/031/L/CNERSH/SP, and 2014/04/440/CE/CNERSH/SP). Participants were enrolled upon signature of an informed consent by their parent or legal guardian.

### Study population

Participants in this study were recruited among asymptomatic children aged from 4–15 years who attended primary schools in the area of Mfou (3°72N; 11°64E), a small city 30 km apart from Yaoundé, Cameroon. A total of seven schools were screened in the area. Parasitological surveys were conducted in collaboration with the medical team of the local hospital during four consecutive seasons of malaria transmission, short rainy seasons (April–May) and long rainy seasons (October–November) in 2013 and 2014.

### Sample collection and parasite quantification

A thick blood smear from finger prick was performed to each volunteer attending school the day of collection. All blood smears were dried, stained with a 10% Giemsa solution for 20 min and then examined microscopically by qualified, experienced microscopists using a Leica^®^ light microscope with a 100× oil-immersion objective. Asexual parasite-positive children were treated with an artemisinin-based combination therapy (ACT) according to national guidelines the day following the parasitological survey. Blood collections for accurate quantification of parasites were performed to those identified as gametocyte carriers. Blood collections from mixed-infections were excluded from the study.

For each gametocyte carrier, thick blood smears were prepared simultaneously in triplicate using a standardized blood volume of 5 µl. Capillary blood (CB) samples were collected by pipetting drops of blood from finger prick dispensed on a clean glass slide. Venous blood (VB), ~200 µl, was drawn from the antecubital vein into a dried Vacutainer^®^ tube pre-warmed in an incubator at 37 °C and 5 µl volumes were pipetted directly from the tube for the microscopy.

Blood smears were treated as described above and slides were blinded prior to microscopic examination, to avoid reading bias due to participant code or blood source. Parasite densities were determined by counting gametocytes against 1000 white blood cells (WBS) and asexual blood stages (ABS) against 500 WBS, assuming a standard number of 8000 WBS/µl of blood.

### Data analysis

The datasets used for statistical analyses are included in Additional files 1 and 2.

Univariate analysis was realized by Chi square and Kruskal–Wallis tests. All 95% confidence intervals for mean were estimated by a bias-corrected and accelerated (BCa) bootstrap procedure (“boot” package). The relationship between sexual and asexual parasite counts was assessed using Spearman’s correlation.

An outcome variable (parasitaemia) was created by averaging individual triplicate measures to smooth a possible within-host variability. Parasite counts were highly skewed and discontinuous, so data were transformed into an ordinal variable (none, low, high). Agreement among paired samples was tested using the Krippendorff’s alpha coefficient [13]. As proposed by Agresti [14], an ordinal response variable is usually analysed by cumulative link models, also known as ordinal regression models. To take into account the pairwise aspect of the data and a possible correlation effect on the dependent variable (parasite density), a cumulative link mixed models (CLMM) was used. Because the study was conducted in seven different sites requiring taking into account local malaria endemicity levels and a possible group effect, the village of inclusion was set as random intercept in each model. To compare the parasitaemia in capillary and venous blood, blood origin measures were adjusted on age and sex. The CLMM was fitted using the Laplace approximation and a probit link function. All analysis was performed in R 3.3.0 [15] with the R package ‘ordinal’ (function ‘clmm’) [16]. To assess whether blood origin had an influence on the response variable, models were compared using Akaike Information Criterion (AIC), the difference in AIC from the top-ranked model (∆AIC) and the blood origin was considered as a significant predictor if P < 0.05.

## Results

### Malaria prevalence in the studied area

The parasitological surveys were conducted over 4 transmission seasons from April 2013 to November 2014. Participants were pupils from 7 primary schools, aged between 4 and 15 years. A total of 4087 individuals were screened for parasite carriage by microscopic examination of thick blood smears from finger pricks. Results from the blood examinations provided data on the extent of the *P. falciparum* asymptomatic reservoir in this high malaria transmission setting. Microscopical readings identified 8.9% (364/4087) gametocyte carriers and their distribution per season, age and school is given in Table 1. The proportion of gametocyte carriers was similar from one season to another (P = 0.116) but varied across schools (P = 0.05; Table 1). *Plasmodium falciparum* asexual parasites were detected in 51.8% (2116/4087) of thick blood smears. Mixed infections were identified for 2.8% (115/4087) of the blood smears. Infections with *P. falciparum* and *Plasmodium malariae* represented 94,8% (109/115) of the mixed infections, *P. falciparum* and *Plasmodium ovale* 4,3% (5 samples) and one blood smear contained the three species. The prevalence of *P. falciparum* gametocytes was not different in mixed species infections and in *P. falciparum* mono-infections (P = 0.84). Presence of *P. falciparum* gametocytes was recorded in 37.1% (135/364) of blood samples that had no *P. falciparum* asexual stages, which gives an overall *P. falciparum* prevalence of 55.1% (2251/4078).

**Table 1:** Malaria prevalence data from the school screenings of asymptomatic individuals (N = 4087)

	N	Gametocyte positive	Asexual parasite positive (%)			
		Pf (%)	Pf	Pm	Po	Mixed
Transmission season						
Short rainy, 2013	766	10.6	54.3	2.1	0.1	1.7
Long rainy, 2013	1086	8.2	51.3	4.7	0.3	3.5
Short rainy, 2014	1008	9.7	49.4	2.6	0.1	1.9
Long rainy, 2014	1227	7.8	52.6	4.5	0.5	3.7
Age						
04–05	980	10.5	55.6	4.4	0.4	3.6
06–10	2129	9.6	53.3	3.9	0.2	2.8
11–15	978	5.8	44.6	2.2	0.2	2.0
Village						
Ekali	1052	7.9	52.8	2.6	0.4	2.3
Ekoko	209	6.2	53.1	6.2	0	4.3
Essazok	259	7.3	49.4	1.9	0.8	1.5
Koumou	419	12.2	47.7	2.6	0	2.1
Metet	213	7.5	51.2	1.9	0	1.9
Nkilzok	742	8.4	52.8	4.2	0.1	3.1
Nkolnda	1193	10.1	57.2	5.3	0.4	3.9

N, number of individuals screened; Pf, *P. falciparum*; Pm, *P. malariae*; Po, *P. ovale*; mixed, mixed species infections

### Comparison of parasite carriage in capillary and venous blood

A total of 137 sets of samples, comprising each 6 blood smears, 3 from capillary blood and 3 from venous blood, were included for the analysis. Coefficient of variation for the three smears was 13.1 for CB and 13.0 for VB within the trophozoite series, and 34.5 for CB and 27.4 for VB within the gametocyte set of data. Participants were pupils from 7 different primary schools, aged between 4 and 15 years. The median age of the participants was 8 years (IQR [6–10]) and 21% were >10 years old. Among the 137 individuals, 71 were males and 66 females (sex-ratio 0.92). The means of parasite densities in capillary and venous blood among asymptomatic infections are presented in Table 2.

**Table 2:** Mean of parasite densities in capillary and venous blood among asymptomatic infections harbouring gametocytes at enrollment (N = 137)

	Capillary blood		Venous blood	
	Gametocyte mean [CI 95%]	Trophozoite mean [CI 95%]	Gametocyte mean [CI 95%]	Trophozoite mean [CI 95%]
Sex				
F	102 [65–236]	5023 [2982–8505]	86 [59–163]	5499 [3277–9194]
M	89 [66–129]	3429 [1817–6 993]	92 [68–139]	3626 [2099–7124]
Age				
04–05	58 [24–118]	10,590 [2061–27,549]	44 [22–85]	7839 [2338–18,672]
06–10	106 [76–190]	4392 [2814–7035]	97 [71–150]	5150 [3 359–8 196]
11–15	72 [50–106]	905 [467–2283]	82 [57–118]	1098 [565–2572]
Village				
Ekali	166 [98–401]	3478 [1678–8854]	141 [90–275]	4050 [2099–9951]
Ekoko	29 [11–57]	574 [101–1379]	30 [11–51]	972 [55–2236]
Essazok	65 [29–112]	653 [142–1954]	61 [28–123]	706 [155–1948]
Koumou	63 [43–94]	3449 [1743–6913]	62 [43–94]	4330 [2065–9074]
Metet	52 [31–78]	8089 [2678–18,980]	58 [35–94]	8626 [3110–20,302]
Nkilzok	75 [42–146]	961 [580–1436]	75 [42–130]	1184 [717–1774]
Nkolnda	110 [56–257]	9179 [3673–20,398]	113 [55–288]	8113 [3203–17,557]

**Fig. 1:**
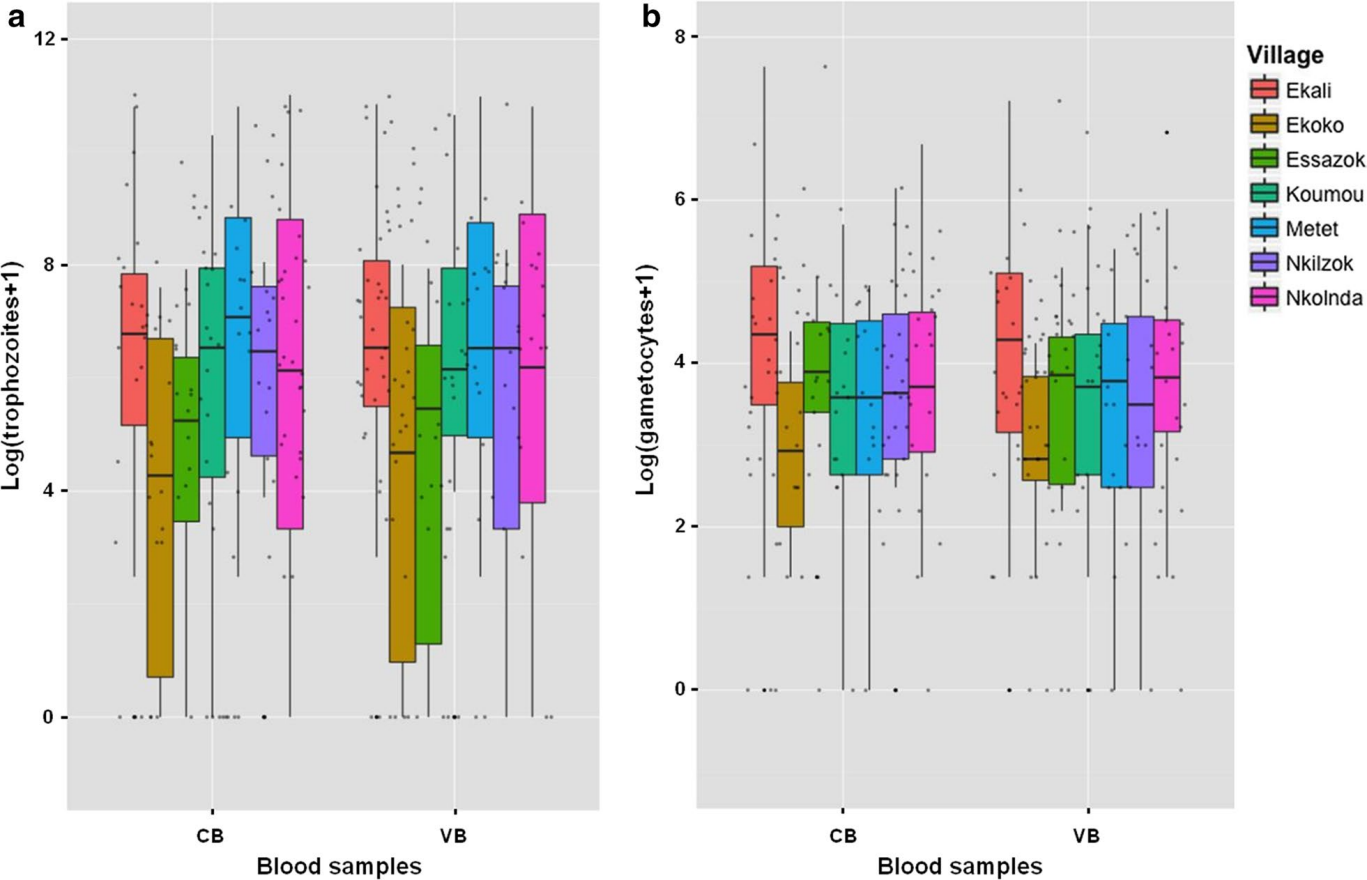
Comparison of *P. falciparum* parasite densities in capillary (CB) and venous blood (VB). **a** Trophozoite densities; **b** gametocyte densities. Data from N = 137 asymptomatic children carrying gametocytes at the time of inclusion and attending seven different schools. Parasite densities are shown on a logarithmic scale to take into account the skewed distribution of parasite counts

**Fig. 2:**
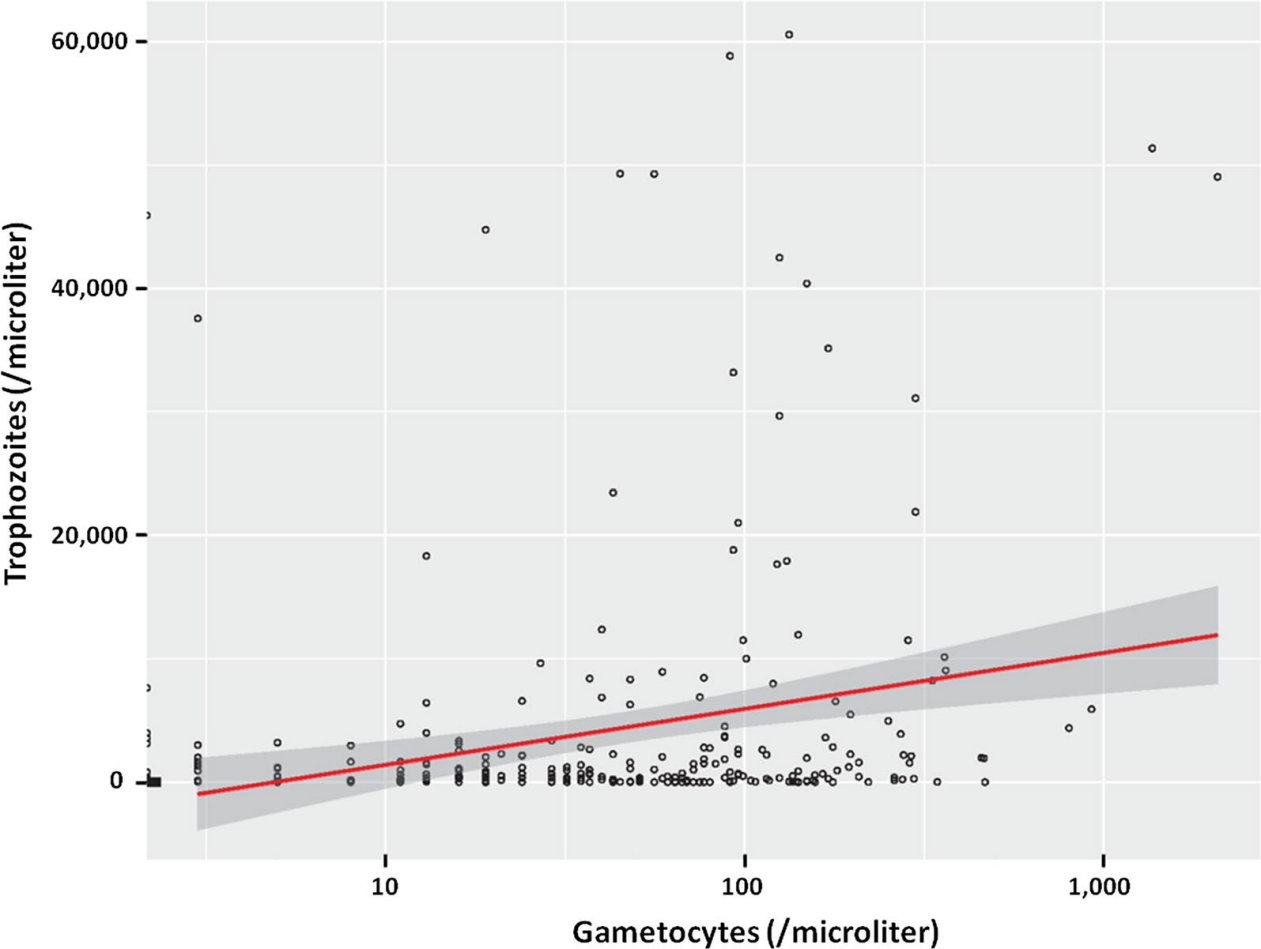
Relationship between gametocyte and trophozoite densities. The *line* indicates the best linear model and the *shaded area* the 95% confidence interval

The Krippendorff’s alpha coefficient was 0.741, indicating good reliability among paired samples. Then further analysis does not distinguish the source of the blood, data from VB and CB were pooled (n = 274). Means of parasite densities varied from 38 to 112 for sexual stage and from 285 to 14,699 for asexual stages. The analysis revealed a positive correlation between the estimated densities of asexual and sexual parasites (Spearman’s rank correlation = 0.162, P = 0.007; Figs. 1 and 2); the density of gametocytes increased as the density of asexual parasites does. Also, the mean parasite densities differed according to the village for both sexual and asexual stages (Fig. 1; Table 2).

Baseline characteristics used in the multivariate ordinal mixed model are presented in Additional file 3 for gametocyte and asexual stage data. Parasite densities were grouped in 3 different classes. For gametocyte data, class 0 corresponds to no gametocyte detected by microscopy. Indeed, at the day of blood collections, 4 (3%) volunteers had no more gametocytes neither in VB nor in CB, 3 (2%) had gametocytes only in one of the VB slides and 4 (3%) only in one CB slide. Results from the CLMM model are summarized in Table 3. There was no effect of blood origin on parasite counts when age, sex and village were considered in the model, not only for gametocytes (P = 0.98) but also for trophozoites (P = 0.47) (Table 3). The “trophozoites” multivariate analysis showed a decrease of parasite densities with increasing age class: asexual densities were halved for the 6-10 years old (P = 0.18) and divided by 3.45 for the 11-15 years old (P = 0.02). A model including “village” as a fixed effect better explained the variation in the parasite densities than a null model without random effect. The significance of the random intercept “village” (P = 0.002) in the trophozoite model (Table 3) indicates different asexual densities between the villages.

**Table 3:** Analysis of blood origin effect on parasitemia measurement adjusted on age, sex and village

	Trophozoite class^a^			Gametocyte class^b^		
	Adjusted			Adjusted		
	P value	OR	CI 95% [2.5–97.5%]	P value	OR	CI 95% [2.5–97.5%]
Sex						
F		1			1	
M	0.31	1.33	[0.77–2.31]	0.98	0.99	[0.49–2.02]
Age^c^						
04–05		1			1	
06–10	0.18	0.52	[0.20–1.36]	0.21	2.22	[0.65–7.60]
11–15	0.02	0.29	[0.10–0.85]	0.08	3.54	[0.88–14.3]
Blood origin						
CB		1			1	
VB	0.47	1.23	[0.72–2.08]	0. 98	1.01	[0.51–2.00]
Village random intercept	0.002			0.45		
Ekali		1.71	[1.05–2.79]		0.72	[0.26–1.19]
Ekoko		0.44	[0.17–1.10]		0.97	[0.54–1.74]
Essazok		0.54	[0.21–1.38]		0.98	[0.54–1.76]
Koumou		1.13	[0.67–1.90]		0.99	[0.62–1.59]
Metet		2.52	[1.34–4.74]		0.72	[0.43–1.23]
Nkilzok		0.52	[0.29–0.95]		0.93	[0.56–1.57]
Nkolnda		1.67	[0.86–3.25]		1.23	[0.73–2.06]

*n* total samples, venous and capillary blood pairs from 137 volunteers

^a^Trophozoite class 0 = [0], class 1 = [1–5000], class 2 = [>5000]

^b^Gametocyte class 0 = [0], class 1 = [1–250], class 2 = [>250]

^c^Age at the time of inclusion

### Gametocyte infectiousness for mosquitoes

Despite repeated efforts, DMFAs with capillary blood were not successful. Membrane feedings were performed using 150 µl of capillary blood for 11 gametocyte donors. The too small amount of blood (400 µl would be required to fill glass feeders) and the technical constraints to collect capillary blood (that coagulates rapidly) did not allow to obtain good results: only 3 assays produced oocysts, but in a small number of mosquitoes: 1 infected mosquito with 1 oocyst out of 25 dissected, 1 mosquito with 3 oocysts/32 dissected and 2 mosquitoes with 1 oocyst/18 dissected.

Prevalence of infection in the 11 experiments was >40% when using venous blood and DMFAs were stopped.

## Discussion

This study aimed to compare densities of gametocyte and asexual stages between capillary and venous blood, and to measure the infectiousness of gametocytes from the different blood sources for mosquitoes.

Unfortunately, it has not been possible to assess whether the source of infectious blood matters for the infectiousness to mosquitoes in DMFAs. Indeed, blood taken from finger pricks was coagulating during collection and the amount required for DMFAs (400 µl) was too large. When using DMFAs to test transmission-blocking vaccine candidates or for the evaluation of transmission-blocking interventions, large numbers of mosquitoes, over 30 mosquitoes per batch, are required to measure the efficacy of different drugs or vaccines, most often at different concentrations, and it will not be possible to obtain the necessary blood amounts (>3 ml) per assay from finger pricks. The major advantage of using capillary blood over venous blood was to avoid venipuncture and any discomfort at the site of puncture. The question about gametocyte infectiousness remains open and further molecular analysis at transcriptomic and proteomic levels may help deciphering whether biological differences exist between gametocytes according to the blood source. The DFMA using venous blood from naturally infected parasite donors and the SMFA that allows mosquito feedings with cultured gametocytes then remains the reference methods to assess the impact of novel transmission-blocking candidates on gametocyte infectivity.

In the present study, malaria parasite densities do not differ between capillary and venous blood in asymptomatic subjects for both gametocyte and trophozoite stages. Previous studies reported that blood films from capillary blood were more sensitive for the detection of asexual stages than that from venous blood [17, 18]. However, Njunda et al. [17] found that parasitaemia among symptomatic patients were not different between the two blood sources. Ouédraogo et al. [18] performed thin blood smears among asymptomatic subjects and found that parasite densities expressed as the number of parasitized red blood cells were higher in capillary blood. But the small sample size and the absence of blood smear replicates in their study suggest a low statistical power. In the present study, blood smears were performed in triplicates and the CLMM model controls the “village” variability, which provides a greater power to confirm there is no difference in parasite densities between the blood sources.

Interestingly, in this study, gametocyte densities were significantly associated with asexual stage parasitaemia. Previous studies reported contrasting results and the relationship between sexual and asexual parasite densities is heterogeneous, depending on the epidemiological setting, age, haematocrit and malaria status (symptomatic versus asymptomatic) [19–22]. Here, asexual parasite densities decreased with age, within the 4–15 age group, but no correlation was observed for age and gametocyte loads, which is in agreement with previous studies conducted in the vicinity of our studied area [23, 24]. Densities of *P. falciparum* asexual stage parasites varied between the screened schools, which is also suggested by significativity (P = 0.002) of the random effect (village) in the trophozoite model (Table 3), and this result may reflect biting heterogeneity, even at small geographical scales. Variation in malaria exposure between villages exists, due to the proximity to larval breeding sites, human behaviour or genetic factors, and it has led to the definition of hotspots of transmission [25–28].

Differences in gametocyte prevalence between the screened schools were observed, but not in the prevalence of *P. falciparum* asexual stages. This could result from a different malaria exposure in the different villages. Indeed, it has been previously reported that gametocyte carriage is higher in people exposed to lower mosquito infectious bites [29, 30]. Also, previous studies in symptomatic subjects reported higher gametocyte carriage in mixed-infections [31]. In this study, prevalence of gametocytes was not different in mixed-infections and in *P. falciparum* mono-infections and this can reflect either a different epidemiological setting or a different relationship in asymptomatic carriers.

Malaria parasite carriage was assessed among pupils from primary schools, one of the most vulnerable populations. A total of 55.1% of participants were infected by *P. falciparum*, a proportion similar to a previous study performed in 2005–2006 in the same area [32]. This result highlights the importance of the asymptomatic reservoir in this studied area and poses challenges to future disease control strategies. Malarial infections were detected by microscopic reading of thick blood smears. This method has theoretical detection limits of 4–10 parasites/µl and it obviously underestimates the real infection prevalence. Indeed, highly sensitive and specific PCR-based methods now permits a detection limit much lower than that of well-trained microscopists [33, 34]. However, molecular screening for parasites is particularly useful to detect submicroscopic infections in areas of low endemicity [22, 35]. In a setting of high malaria transmission intensity such as in our studied area, submicroscopic infections contribute less to mosquito transmission [36]. Nonetheless the development of reliable molecular methods for the specific detection of mature gametocytes would be helpful to the implementation of DMFAs at a larger scale in laboratories from malaria endemic areas.

## Conclusion

This study was conducted to better understand the variability observed in DMFAs and investigated differences in the gametocyte concentration between microvasculature and vein. No significant differences in the gametocyte densities between capillary and venous blood was detected and this result suggests that the blood source, capillary or venous, should not interfere with transmission efficiency in membrane feeding experiments.

## Additional files

**Additional file 1.** Sample information and associated Plasmodium prevalence.

**Additional file 2.** Sample information, associated parasite densities and corresponding class.

**Additional file 3: Table S1.** Baseline characteristics at the parasite class level.
