# Supplementary material for: Do the venous blood samples replicate malaria parasite densities found in capillary blood? A field study performed in naturally-infected asymptomatic children in Cameroon
Source: Malar J. 2017 Aug 17;16:345. doi: 10.1186/s12936-017-1978-6 (PMC5561596; doi:10.1186/s12936-017-1978-6)
Supplement: Supplementary file 3 — Additional file 3: Table S1. Baseline characteristics at the parasite class level. [file 12936_2017_1978_MOESM3_ESM.docx]

Additional Table 1: Baseline characteristics at the parasite class level (n = 274)

|  |  |  | **Trophozoite Class^*^** | | |  |  |  | **Gametocyte Class**^§^ | | |  |  |
| --- | --- | --- | --- | --- | --- | --- | --- | --- | --- | --- | --- | --- | --- |
|  | **Total** |  | **0** | **1** | **2** |  | **P-value** |  | **0** | **1** | **2** |  | **P-value** |
| Sex | | | | | | | |  |  | | | | |
| F | 132 (48%) |  | 20 (15%) | 87 (66%) | 25 (19%) |  | 0.056 |  | 8 (6%) | 113 (86%) | 11 (8%) |  | 0.96 |
| M | 142 (52%) |  | 11 (8%) | 111 (78%) | 20 (14%) |  |  |  | 7 (5%) | 123 (87%) | 12 (8%) |  |  |
| Age^†^ | | | | | | | |  |  | | | | |
| 04-05 | 24 (9%) |  | 3 (12%) | 14 (58%) | 7 (29%) |  | 0.01 |  | 3 (12%) | 20 (83%) | 1 (4%) |  | 0.12 |
| 06-10 | 192 (70%) |  | 21 (11%) | 135 (70%) | 36 (19%) |  |  |  | 12 (6%) | 163 (85%) | 17 (9%) |  |  |
| 11-15 | 58 (21%) |  | 7 (12%) | 49 (84%) | 2 (3%) |  |  |  | 0 (0%) | 53 (91%) | 5 (9%) |  |  |
| Blood origin | | | | | | | |  |  | | | | |
| CB | 137 (50%) |  | 16 (12%) | 101 (74%) | 20 (15%) |  | 0.76 |  | 7 (5%) | 119 (87%) | 11 (8%) |  | 0.93 |
| VB | 137 (50%) |  | 15 (11%) | 97 (71%) | 25 (18%) |  |  |  | 8 (6%) | 117 (85%) | 12 (9%) |  |  |
| Village | | | | | | | |  |  | | | | |
| Ekali | 70 (26%) |  | 3 (4%) | 54 (77%) | 13 (19%) |  | < 0.0001 |  | 3 (4%) | 57 (81%) | 10 (14%) |  | 0.096 |
| Ekoko | 12 (4%) |  | 4 (33%) | 8 (67%) | 0 (0%) |  |  |  | 0 (0%) | 12 (100%) | 0 (0%) |  |  |
| Essazok | 12 (4%) |  | 3 (25%) | 9 (75%) | 0 (0%) |  |  |  | 0 (0%) | 12 (100%) | 0 (0%) |  |  |
| Koumou | 66 (24%) |  | 8 (12%) | 47 (71%) | 11 (17%) |  |  |  | 3 (5%) | 59 (89%) | 4 (6%) |  |  |
| Metet | 34 (12%) |  | 0 (0%) | 24 (71%) | 10 (29%) |  |  |  | 4 (12%) | 30 (88%) | 0 (0%) |  |  |
| Nkilzok | 42 (15%) |  | 7 (17%) | 35 (83%) | 0 (0%) |  |  |  | 5 (12%) | 32 (76%) | 5 (12%) |  |  |
| Nkolnda | 38 (14%) |  | 6 (16%) | 21 (55%) | 11 (29%) |  |  |  | 0 (0%) | 34 (89%) | 4 (11%) |  |  |

n: total samples, venous and capillary blood pairs from 137 volunteers
*Class 0 = [0], Class 1 =[1-5 000], Class 2 =[>5 000]

^§^ Gametocyte Class 0 = [0], Class 1 = [1-250], Class 2 = [>250]
^†^ Age at the time of inclusion.
